# Fitness comparison of *Plutella xylostella* on original and marginal hosts using age‐stage, two‐sex life tables

**DOI:** 10.1002/ece3.7804

**Published:** 2021-06-23

**Authors:** Fei‐Ying Yang, Jun‐Hui Chen, Qian‐Qian Ruan, Bei‐Bei Wang, Lu Jiao, Qing‐Xuan Qiao, Wei‐Yi He, Min‐Sheng You

**Affiliations:** ^1^ State Key Laboratory for Ecological Pest Control of Fujian and Taiwan Crops Institute of Applied Ecology Fujian Agriculture and Forestry University Fuzhou China; ^2^ International Joint Research Laboratory of Ecological Pest Control Ministry of Education Fujian Agriculture and Forestry University Fuzhou China; ^3^ Key Laboratory of Integrated Pest Management for Fujian‐Taiwan Crops Ministry of Agriculture Fuzhou China; ^4^ Institute of Microbiology Jiangxi Academy of Sciences Nanchang China; ^5^ Xiaoshan Agricultural Technology Extension Center Hangzhou China

**Keywords:** adaptation, diamondback moth, fitness, host shift, pea, radish

## Abstract

The diamondback moth, *Plutella xylostella*, is an important agricultural pest that severely damages cruciferous vegetables. Although previously considered a threat only to *Brassica* species, *P. xylostella* has been observed to feed on noncruciferous vegetables. Here, we established a population of *P. xylostella* on the pea *Pisum sativum* (PxP population). We compared this PxP population's performance on the pea host plant to a population (PxR) reared on the original host plant radish (*Raphanus sativus*) for several generations using an age‐stage, two‐sex life table and analyzed the correlations between different fitness parameters. In the 1st generation of the PxP population, survival rate of immature stage was 17%, while the survival rate of PxR was 68%; the duration of the 4th larval instar (5.30 d) and mortality (25%) of this generation were significantly longer (2.8 d) and higher (1%) than that of PxR, respectively (both *p* < .001). Upon long‐term acclimation, the PxP fitness improved significantly, especially that the survival rate of immature stages increased to approximately 60% in the 15th, 30th, and 45th generations. However, PxP feeding on pea exhibited poorer fitness with longer larval developmental time, shorter total life span, lighter pupa, and lower fecundity in different generations compared with PxP feeding on radish. PxP feeding on pea also showed a significantly lower intrinsic rate of increase (*r*), net reproduction rate (*R*_0_), finite increase rate (*λ*), and longer mean generation time (*T*) than PxP feeding on radish in all generations tested. Significant positive correlations were observed between pupal weight and female fecundity in pea‐fed populations, and between female longevity and female fecundity in pea‐fed and radish‐fed populations. Our findings suggest that *P. xylostella* adaptation to pea does not improve overall fitness compared with the original host radish, making pea a marginal host for *P. xylostella*.

## Graphical abstract

We compared the performance of diamondback moth (*Plutella xylostella*) feeding on the original host plant radish (*Raphanus sativus*) and the marginal host plant pea (*Pisum sativum*). We found that the *P. xylostella* fitness was significantly improved upon long‐term acclimation on pea and the correlation between different fitness parameters was influenced by host plants.

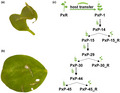

## 1 INTRODUCTION

The diamondback moth, *Plutella xylostella* (Lepidoptera: Plutellidae), is an economically significant pest, jeopardizing various vegetables in the Brassicaceae family and causing total yield losses and pest management costs of US$4‐5 billion annually worldwide (Zalucki et al., 2012). The pest status of *P. xylostella* has increased on Brassicaceae crops in different parts of the world recently (Furlong et al., 2013), likely due to its short life cycle, resistance to various insecticide classes, and adaptability to different environments, and the increasing agricultural demand of *Brassica* vegetables and oilseed crops (Furlong et al., 2013; Li et al., 2016; Talekar & Shelton, 1993). It is mainly considered an oligophagous pest, only feeding on Brassicaceae plants. However, its dietary behavior has been questioned. For example, Löhr and Gathu (2002) found a field of sugar snap pea, *Pisum sativum* (Fabaceae) being destroyed by a *P. xylostella* population, in Kenya, indicating polyphagous behavior. This population adapted quickly to peas under laboratory conditions, with larval survival rate increasing from 2.4% in the 1st generation to 49.7% in the 4th generation (Löhr & Gathu, 2002). The mean generation time of *P. xylostella* also showed a negative trend over successive generations (Henniges‐Janssen et al., 2011), indicating adaptability to pea. However, other population parameters of *P. xylostella* feeding on pea remain unknown.

An age‐stage, two‐sex life table is designed to study the demographics of an insect population in response to environmental variables, including host plant (Chi et al., 2020). Population parameters such as intrinsic rate of increase (*r*), finite rate of increase (*λ*), net reproductive rate (*R*_0_), and mean generation time (*T*) describe characteristics of a population as values. These are effective estimators to predict the potential insect population sizes on different host plants. Guo et al. (2021) used an age‐stage, two‐sex life table to compare the population fitness of *Spodoptera frugiperda* populations feeding on maize, tobacco, and potato. Golizadeh et al. (2009a) compared the life tables of *P. xylostella* feeding on five different cultivated *Brassica* host plants. Fathipour et al. (2019) and Soufbaf et al. (2010) compared the performance of *P. xylostella* on five and ten canola cultivars, respectively. Nikooei et al. (2015) compared *P. xylostella* performance on different genetically manipulated *Brassica* plants (canola's progenitor, two cultivated canola cultivars, one hybrid, one gamma‐ray mutant, and one transgenic genotype).

Extensive studies have reported the correlations between various fitness parameters among insect populations. Development time was correlated with body size in insects, but these correlations could be positive, zero, or negative (Nijhout et al., 2010; Teder et al., 2014). Fecundity and body weight or developmental rate were positively correlated in 60 insect species from eight orders, including Coleoptera and Lepidoptera (Calvo & Molina, 2005; Coyle et al., 1999). In addition, studies showed that diet can change the correlations between these parameters (Gebhardt & Stearns, 1988). For example, the nutrient quantity and quality of insect foods could influence the correlation between development time and body size (Kause et al., 2001; Uhl et al., 2004). However, whether these correlations apply to *P. xylostella* feeding on different host plants remains unknown.

The objective of this study was to compare the population demographics of *P. xylostella* after long‐term acclimation to its marginal host plant, pea, and to its original host plant, radish. Using an age‐stage, two‐sex life table, we examined the life history parameters of survivorship, development time, and fecundity. We compared these parameters between different generations of *P. xylostella* feeding on radish and on pea, and studied the correlations between pupal weight and female fecundity, pupal weight and adult longevity, duration of larval stage and pupal weight, and female longevity and female fecundity.

## 2 MATERIALS AND METHODS

### 2.1 Insects and plants

*Plutella xylostella* pupae were initially collected from *Brassica* fields in July 2004 in the Fuzhou suburban area (26.08°N, 119.28°E) and reared in the laboratory on “Nanpanzhou” cultivar radish (*Raphanus sativus*) for more than 200 generations (referred to as PxR population). To establish a PxP population, PxR eggs were artificially placed on “Purple Flower‐Soft Pod” cultivar pea (*Pisum sativum*) leaves. We named the 1st generation of *P. xylostella* after the host shift as PxP‐1, and so on in a similar fashion (PxP‐n). Upper generations of the PxP population feeding on radish were named PxP‐n_R. Larvae were left to feed on radish or pea under controlled conditions of 23 ± 1°C, 65 ± 5% relative humidity (RH), and 16‐hr light: 8‐hr dark photoperiod. Adults were provided with a 10% w/v honey–water mixture.

Radish was cultivated in rectangular plastic trays (420 × 320 × 100 mm) with nutrition soil, and pea was cultivated in disposable nutrition bags (16 × 14 mm) with peat soil. Plants were kept in fine transparent cages with 0.1 mm mesh screens in a walk‐in growth chamber at 23 ± 1°C and 65 ± 5% RH, under a 16‐hr light: 8‐hr dark photoperiod. Leaves of radish at 1 week and pea at 4 weeks were used to feed *P. xylostella* larvae.

### 2.2 Investigation of fitness parameters

**FIGURE 1:**
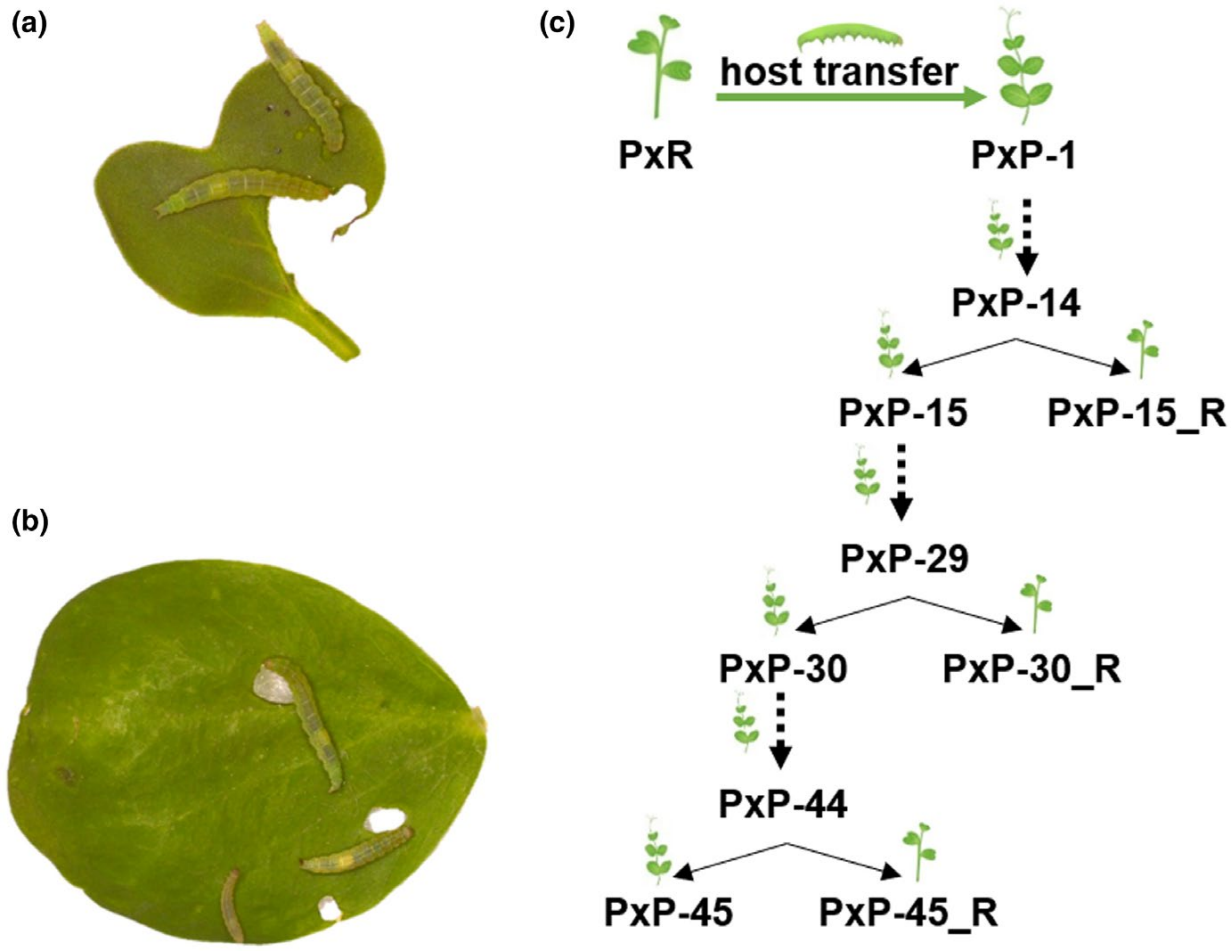
*Plutella xylostella* feeding on radish (a) and pea (b), and schematic diagram of experimental design (c). PxR, *P. xylostella* reared on radish; PxP‐1, PxP‐14, PxP‐15, PxP‐29, PxP‐30, PxP‐44, and PxP‐45: the 1st, 14th, 15th, 29th, 30th, 44th, and 45th generations of PxP (*Plutella xylostella* reared on pea) population; PxP‐15_R, PxP‐30_R, and PxP‐45_R: the 15th, 30th, and 45th generations of PxP population feeding on radish

The development time, survival, and reproduction of *P. xylostella* feeding on radish and pea leaves were investigated and compared. The eggs of the PxR population and the 14th, 29th, and 44th generations of the PxP population were kept separately on radish and on pea, and life tables (PxR, PxP‐1, PxP‐15, PxP‐15_R, PxP‐30, PxP‐30_R, PxP‐45, and PxP‐45_R) were studied (Figure 1). One hundred newly laid (<2 hr) light yellow eggs were placed in plastic film dishes measuring 9 cm in diameter. The neonates were counted and individually transferred to film dishes measuring 60 mm in diameter, with fresh radish or pea leaves. Fresh leaves were provided daily, and uneaten leaves were removed. The survival and development times of each developmental stage were recorded daily. One pair of newly emerged female and male moths was confined in a plastic chamber (29 × 38 × 32 mm) capped by a fine mesh for ventilation. Chambers were lined with film papers with grooves serving as oviposition substrates. Eggs laid by each female moth were counted daily until it died. Parameters such as survivorship, fecundity, oviposition period, and total life span were recorded. Pupal weight was also recorded at the second day after pupation with 0.1 mg measurement accuracy (OHAUS CORPORATION^®^ AR224CN balance, China). Each newly hatched larva was considered as one replicate.

### 2.3 Data analysis

Life history data of PxR and PxP populations were analyzed using TWOSEX‐MSChar (V2018.05.04) (Chi, 2019; Chi & Liu, 1985; Chi et al., 2020). The age‐stage survival rate (*S_xj_*) was calculated based on the age‐stage‐structure matrix (Chi & Liu, 1985). The formula for the parameters was calculated as follows:

$$l_{x}=\sum_{j=1}^{m}s_{\mathrm{xj}}$$

$$m_{x}=\frac{\sum_{j=1}^{m}s_{\mathrm{xj}}f_{\mathrm{xj}}}{\sum_{j=1}^{m}s_{\mathrm{xj}}}$$

$$R_{0}=\sum_{x=0}^{\omega }l_{x}m_{x}$$

$$\sum_{x=0}^{\omega }e^{‐\gamma (x+1)}l_{x}m_{x}=1$$

$$\lambda =e^{\gamma }$$

$$T=\frac{l_{n}R_{0}}{\gamma }$$

where *l_x_* is age‐specific survival rate, *f_xj_* is age‐stage specific fecundity, *m_x_* is age‐specific fecundity, *R*_0_ is net reproductive rate, *r* is intrinsic rate of increase, *λ* is finite rate, and *T* is mean generation time.

The variances and standard errors of these life history parameters were calculated 100,000 times using the bootstrap technique. Data for each parameter were analyzed separately using paired bootstrap tests, except for pupal weight, which was analyzed using one‐way ANOVA followed by Tukey's test.

Pearson correlation coefficients were used to analyze the correlations between different fitness parameters (pupal weight and female fecundity, pupal weight and adult longevity, duration of larval stage and pupal weight, and female longevity and female fecundity) using packages ggplot2, ggpubr, and ggpmisc in the R software environment (V3.6.3; R Core Team, 2017). SigmaPlot (V12.0) was used for creating scientific graphs.

## 3 RESULTS

### 3.1 Development, survivorship, and reproduction

The development time for each immature stage, adult longevity, total life span, pupal weight, and female fecundity of PxP feeding on radish and on pea in different generations are shown in Table 1. In the 1st generation of the PxP population, only 49 neonates out of 100 eggs successfully developed to the 2nd instar, compared with 82 in the PxR population. The duration of the 4th instar of PxP‐1 (5.30 d) was longer than that of PxR (2.80 d) (*p* < .001). The mortality rate of PxP‐1 was significantly higher (25%) than that of PxR (1%) (*p* < .001). The duration of the larval stage from the 1st to 4th instar was significantly shorter in radish‐fed groups than in pea‐fed groups (all *p* < .05). The duration of the pupa stage of PxP larvae feeding on pea in the 30th (5.61 d) and 45th (4.53 d) generations was longer than those of larvae feeding on radish (5.28 d and 4.15 d, respectively). Preadult duration, including larval and pupal duration, of PxP feeding on pea or radish were significantly shorter in higher generations than in lower generations (all *p* < .001). Generally, the longevity of female or male moths showed no differences between radish and pea diets; for example, the female and male longevity were 8.65 d and 11.72 d in pea in the 45th generation, compared with 8.87 d and 12.86 d in radish (both *p* > .05), respectively. Female longevity of PxP feeding on pea or radish fluctuated in different generations, with the highest values observed in PxP‐15 (12.83 d, 14.21 d, respectively), but male longevity showed no significant differences across generations (all *p* > .05). The total life span of PxP feeding on radish showed a decreased trend from 36.76 d in the 1st generation to 34.92 d, 30.70 d and 26.53 d in the 15th, 30th, and 45th generations in radish, respectively; the total life span of PxP feeding on pea fluctuated from 36.35 d in the 1st generation to 38.42 d in the 15th generation, 29.00 d in the 30th generation, and 25.01 d in the 45th generation.

**TABLE 1:** Developmental time, longevity, pupal weight, and mean fecundity of PxP population (*Plutella xylostella* reared on pea) on radish and on pea in different generationss

Parameter	Host plant	PxP‐1/PxR		PxP‐15/PxP‐15_R		PxP‐30/PxP‐30_R		PxP‐45/PxP‐45_R	
		*n*	Mean ± *SE*	*n*	Mean ± *SE*	*n*	Mean ± *SE*	*n*	Mean ± *SE*
Larva (d)	Pea	27	15.44 ± 0.58Aa	69	12.42 ± 0.33Ab	62	12.27 ± 0.25Ab	71	8.58 ± 0.09Ac
	Radish	74	12.72 ± 0.21Ba	58	11.28 ± 0.34Bb	63	9.10 ± 0.17Bc	65	7.11 ± 0.08Bd
1st instar (d)	Pea	49	3.73 ± 0.17Aa	73	3.59 ± 0.14Aa	75	2.73 ± 0.10Ab	85	2.07 ± 0.08Ac
	Radish	82	4.01 ± 0.13Aa	75	3.11 ± 0.10Bb	74	2.15 ± 0.06Bc	84	1.96 ± 0.05Ad
2nd instar (d)	Pea	39	3.46±0.17Aa	72	2.68±0.16Abc	68	2.94±0.14Ab	79	2.47±0.08Ac
	Radish	77	3.17±0.12Aa	63	2.57±0.18Ab	72	1.92±0.12Bc	69	1.93±0.04Bc
3rd instar (d)	Pea	36	3.03±0.14Aa	70	2.59±0.10Ab	65	2.56±0.20Ab	75	1.77±0.07Ac
	Radish	75	2.87±0.11Aa	59	2.81±0.20Aa	64	2.03±0.08Bb	66	1.47±0.07Bc
4th instar (d)	Pea	27	5.30±0.38Aa	69	3.70±0.21Ab	62	4.06±0.16Ab	71	2.31±0.06Ac
	Radish	74	2.80±0.07Bb	58	2.91±0.11Bab	63	3.16±0.11Ba	65	1.77±0.07Bc
Pupa (d)	Pea	17	5.94±0.16Aa	62	5.32±0.10Ab	54	5.61±0.10Aa	62	4.53±0.08Ac
	Radish	68	5.68±0.06Aa	55	5.20±0.12Ab	60	5.28±0.07Bb	60	4.15±0.08Bc
Preadult duration (d)	Pea	17	24.35±0.69Aa	62	21.47±0.30Ab	54	21.67±0.24Ab	62	16.10±0.12Ac
	Radish	68	22.00±0.23Ba	55	20.24±0.34Bb	60	18.23±0.18Bc	60	14.22±0.09Bd
Adult longevity (d)	Pea	17	12.41±2.31Aab	62	13.45±0.91Ba	54	9.04±0.75Ab	62	10.44±0.66Ab
	Radish	68	14.35±1.12Ab	55	18.18±1.09Aa	60	10.77±0.64Ac	60	10.80±0.65Ac
Female longevity (d)	Pea	9	8.89±2.05Aab	30	12.83±1.00Aa	25	8.76±0.58Ab	26	8.65±3.91Ab
	Radish	30	9.33±0.79Ab	29	14.21±1.20Aa	32	9.91±0.69Ab	31	8.87±0.73Ab
Male longevity (d)	Pea	8	16.38±4.11Aa	32	14.03±1.50Ba	29	9.28±1.31Aa	36	11.72±0.95Aa
	Radish	38	18.32±1.66Aa	26	22.62±1.46Aa	28	11.75±1.1Ab	29	12.86±0.98Ab
Total life span (d)	Pea	17	36.76±2.12Aa	62	34.92±0.92Ba	54	30.70±0.80Ab	62	26.53±0.68Ac
	Radish	68	36.35±1.19Aa	55	38.42±1.07Aa	60	29.00±0.63Ab	60	25.02±0.67Ac
Pupal weight (mg)	Pea	25	4.32±1.27Ba	69	4.48±1.10Ba	62	4.35±0.01Ba	69	4.09±0.08Ba
	Radish	75	6.86±0.97Aa	58	6.05±0.96Ab	60	5.83±0.12Ab	62	5.38±0.11Ac
Female fecundity (eggs/female)	Pea	9	57.33±19.55Bb	30	94.83±8.55Ba	25	68.16±9.189Bb	26	76.15±6.40Bab
	Radish	30	173.20±11.40Aa	29	143.03±13.42Aab	32	131.28±10.40Ab	31	121.29±9.12Ab

Note

Different capital letters within a separate column indicate significant differences between different host plants in the same generation and different lowercase letters within a row indicate significant differences between different generations in the same host plant using the paired bootstrap test (*p* < .05), while the same letters represent no significant difference. One‐way ANOVA followed by Tukey's test was used to analyze pupal weight. PxR, *P. xylostella* reared on radish; PxP‐1, PxP‐15, PxP‐30, and PxP‐45: the 1st, 15th, 30th, and 45th generations of PxP population; PxP‐15_R, PxP‐30_R, and PxP‐45_R: the 15th, 30th, and 45th generations of PxP population feeding on radish.

The average pupal weight of PxP feeding on pea was significantly lower than that of PxP feeding on radish in all generations (4.32 mg vs. 6.86 mg in the 1st generation, 4.48 mg vs. 6.05 mg in the 15th generation, 4.35 mg vs. 5.83 mg in the 30th generation, and 4.09 mg vs. 5.38 mg in the 45th generation) (all *p* < .001). No generational difference was observed in pupal weight of PxP feeding on pea (all *p* > .05), while a negative generational trend was observed in pupal weight of PxP feeding on radish. The mean female fecundities of *P. xylostella* feeding on radish were significantly higher than those on pea in all generations (173.20 eggs vs. 57.33 eggs in the 1st generation, 143.03 eggs vs. 94.83 eggs in the 15th generation, 131.28 eggs vs. 69.16 eggs in the 30th generation, and 121.29 eggs vs. 76.15 eggs in the 45th generation) (all *p* < .01). The mean female fecundities of PxP feeding on pea fluctuated with generations, with PxP‐15 exhibiting the highest value (94.83 eggs). Meanwhile, the mean female fecundities of PxP feeding on radish showed an inverse correlation with generation.

### 3.2 Age‐stage survival rate and fecundity

**FIGURE 2:**
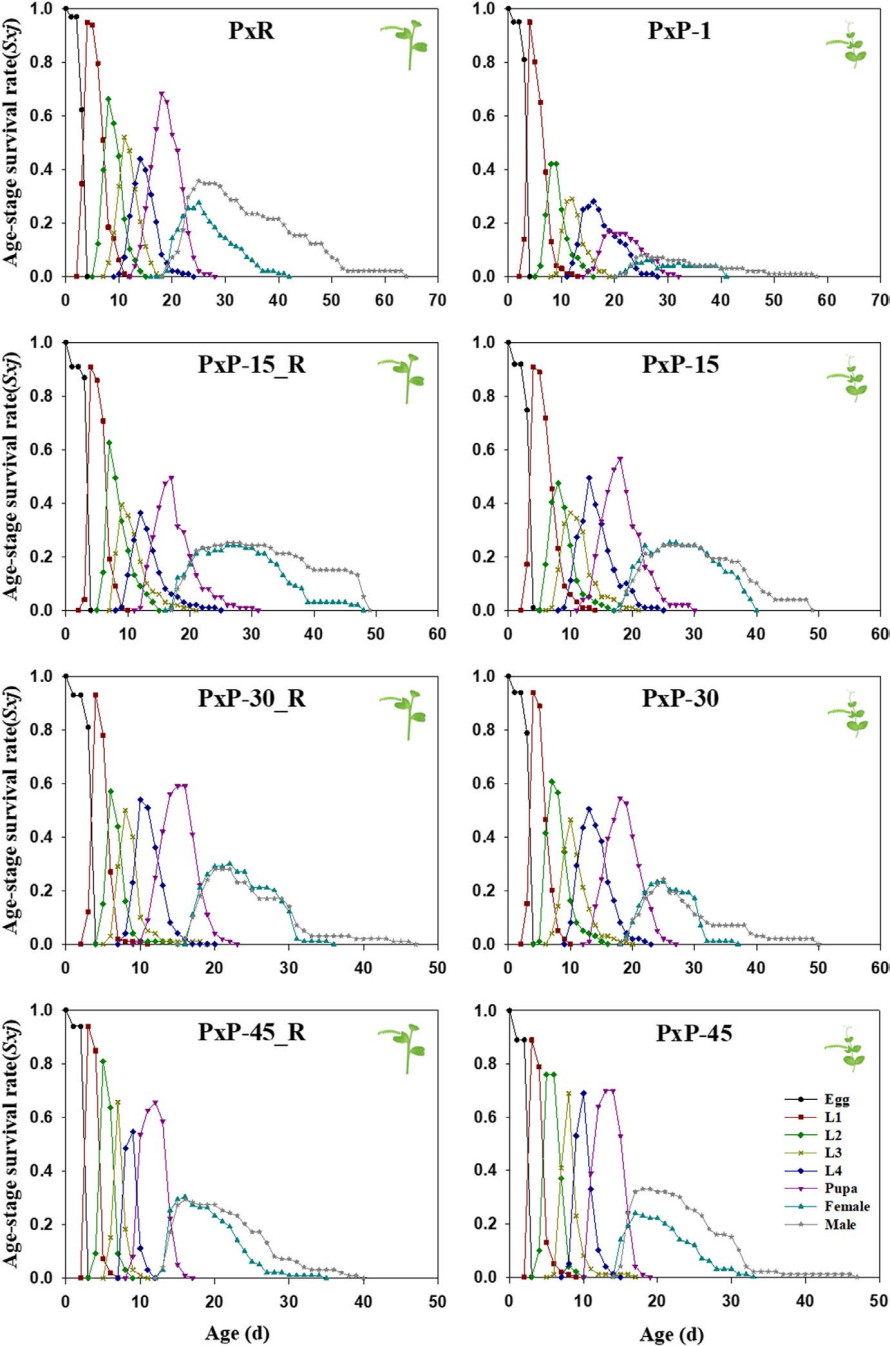
Age‐stage survival rates (*S_xj_*) of PxP population (*Plutella xylostella* reared on pea) feeding on radish and pea in different generations. L1, 1st instar; L2, 2nd instar; L3, 3st instar; L4, 4th instar. PxR, *P. xylostella* reared on radish; PxP‐1, PxP‐15, PxP‐30, and PxP‐45: the 1st, 15th, 30th, and 45th generations of PxP population; PxP‐15_R, PxP‐30_R, and PxP‐45_R: the 15th, 30th, and 45th generations of PxP population feeding on radish

The age‐stage survival rate (*S_xj_*), defined as the survivorship to age *x* and stage *j,* was plotted in Figure 2. A mortality rate of nearly 60% was observed in the 1st instar larvae of the 1st generation of the PxP population. Newly hatched larvae of *P. xylostella* exhibited higher survival rate of immature stages, with 69.4% survival on radish, versus 17.0% on pea plants in the 1st generation. *Plutella xylostella* gradually adapted to pea, with about 60% survival in the 15th, 30th, and 45th generations. The PxP population exhibited similar survival in its original host plant radish, with 55.6% survival rate of immature stages in the 15th generation, 60% of immature stages in the 30th generation, and 60.6% of immature stages in the 45th generation.

**FIGURE 3:**
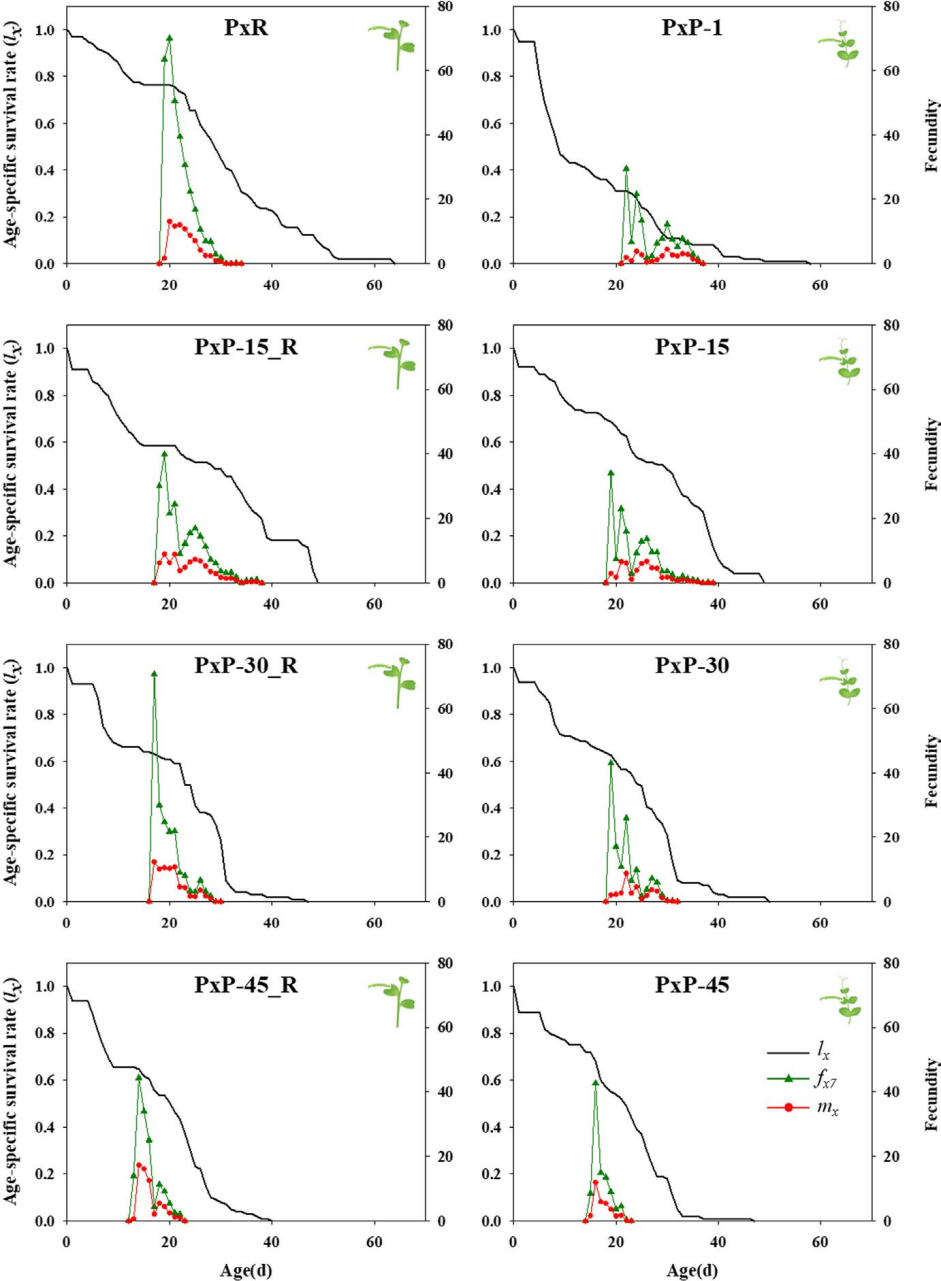
Age‐specific survival rates (*l_x_*), female age‐stage specific fecundity (*f_xj_*), and age‐specific fecundity of total population (*m_x_*) of PxP population (*Plutella xylostella* reared on pea) feeding on radish and pea in different generations. PxR, *P. xylostella* reared on radish; PxP‐1, PxP‐15, PxP‐30, and PxP‐45: the 1st, 15th, 30th, and 45th generations of PxP population; PxP‐15_R, PxP‐30_R, and PxP‐45_R: the 15th, 30th, and 45th generations of PxP population feeding on radish

The observed age‐specific survival rates (*l_x_*), the age‐stage specific fecundity (*f_xj_*), and age‐specific fecundity (*m_x_*) are shown in Figure 3. The *l_x_* curve, describing the change in survival rate of the population with age, showed that PxP feeding on pea exhibited low survivorship in the 1st instar and a short life span in the 1st generation, but gradually improved larval survivorship of different instars at later generations. The *f_xj_* curve, describing the daily number of eggs produced per female of age *x* and stage *j*, only had a single curve *f_x7_* (female stage) because only females reproduce. The highest daily fecundities of *P. xylostella* on the pea and radish were 29.5 eggs and 70.0 eggs in the 1st generation, 34.0 eggs and 39.8 eggs in the 15th generation, 43.0 eggs and 70.63 eggs in the 30th generation, and 42.7 eggs and 44.4 eggs in the 45th generation, which occurred at the age 22nd d and 20th d, 19th d and 19th d, 19th d and 17th d, 16th d and 14th d, respectively. The *m_x_* curve, describing the start times and duration of the reproductive phase, began at age 22nd d in PxP feeding on pea in the 1st generation, which was 3 days later than that of PxR. In addition, the maximal daily oviposition rate of PxP feeding on pea occurred at an average age of 30th d, with mean fecundity of 4.45 eggs per female, which was lower than that of PxR (20th d, 13.07 eggs per female). In the later generations, the duration, reproduction, and maximal daily oviposition showed no evident difference between the two host plants.

### 3.3 Population parameters

The intrinsic rates of increase (*r*), finite rates of increase (*λ*), net reproductive rates (*R*_0_), and mean generation times (*T*) for various groups are shown in Table 2. The *r*, *λ*, and *R*_0_ values of PxP feeding on radish were significantly higher than those of PxP feeding on pea in all generations (all *p* <.05). In addition, the observed *T* values were significantly shorter in PxP feeding on radish than in PxP feeding on pea in all generations (all *p* < .05). The *r*, *λ*, and *R*_0_ values of PxP feeding on pea or on radish in high generations were significantly higher than those in the 1st generation (all *p* < .05), but the *R*_0_ values of PxP feeding on radish showed no difference across generations. In addition, the *T* values were significantly lower in high generations of the PxP population than in lower generations (all *p* < .05).

**TABLE 2:** Population parameters of PxP population (*Plutella xylostella* reared on pea) on radish and on pea in different generations

Parameter	Host plant	PxP‐1/PxR	PxP‐15/PxP‐15_R	PxP‐30/PxP‐30_R	PxP‐45/PxP‐45_R
*r* (d^−1^)	Pea	0.059 ± 0.020Bc	0.136 ± 0.007Bb	0.120 ± 0.010Bb	0.166 ± 0.011Ba
	Radish	0.171 ± 0.008Ab	0.161 ± 0.009Ab	0.183 ± 0.009Ab	0.222 ± 0.011Aa
*λ* (d^−1^)	Pea	1.061 ± 0.021Bc	1.146 ± 0.009Bb	1.127 ± 0.011Bb	1.181 ± 0.013Ba
	Radish	1.187 ± 0.009Ab	1.175 ± 0.010Ab	1.200 ± 0.011Ab	1.248 ± 0.013Aa
*R*_0_ (eggs/female)	Pea	5.160 ± 2.32Bb	28.737 ± 5.076Ba	17.210 ± 3.752Ba	19.800 ± 3.728Ba
	Radish	53.020 ± 8.78Aa	41.899 ± 7.645Aa	42.010 ± 6.97Aa	37.980 ± 6.33Aa
*T* (d)	Pea	27.770 ± 1.580Aa	24.698 ± 0.466Aab	23.740 ± 0.369Ab	17.972 ± 0.169Ac
	Radish	23.170 ± 0.26Ba	23.207 ± 0.474Ba	20.456 ± 0.217Bb	16.418 ± 0.172Bc

Note

Different capital letters within a separate column indicate significant differences between different host plants in the same generation and different lowercase letters within a row indicate significant differences between different generations in the same host plant using the paired bootstrap test (*p* < .05), while the same letters represent no significant difference. PxR, *P. xylostella* reared on radish; PxP‐1, PxP‐15, PxP‐30, and PxP‐45: the 1st, 15th, 30th, and 45th generations of PxP population; PxP‐15_R, PxP‐30_R, and PxP‐45_R: the 15th, 30th, and 45th generations of PxP population feeding on radish. *r*, intrinsic rate of increase; *λ*, finite rate; *R*_0_, net reproduction rate; *T*, mean generation time.

### 3.4 Correlation between fitness parameters

**FIGURE 4:**
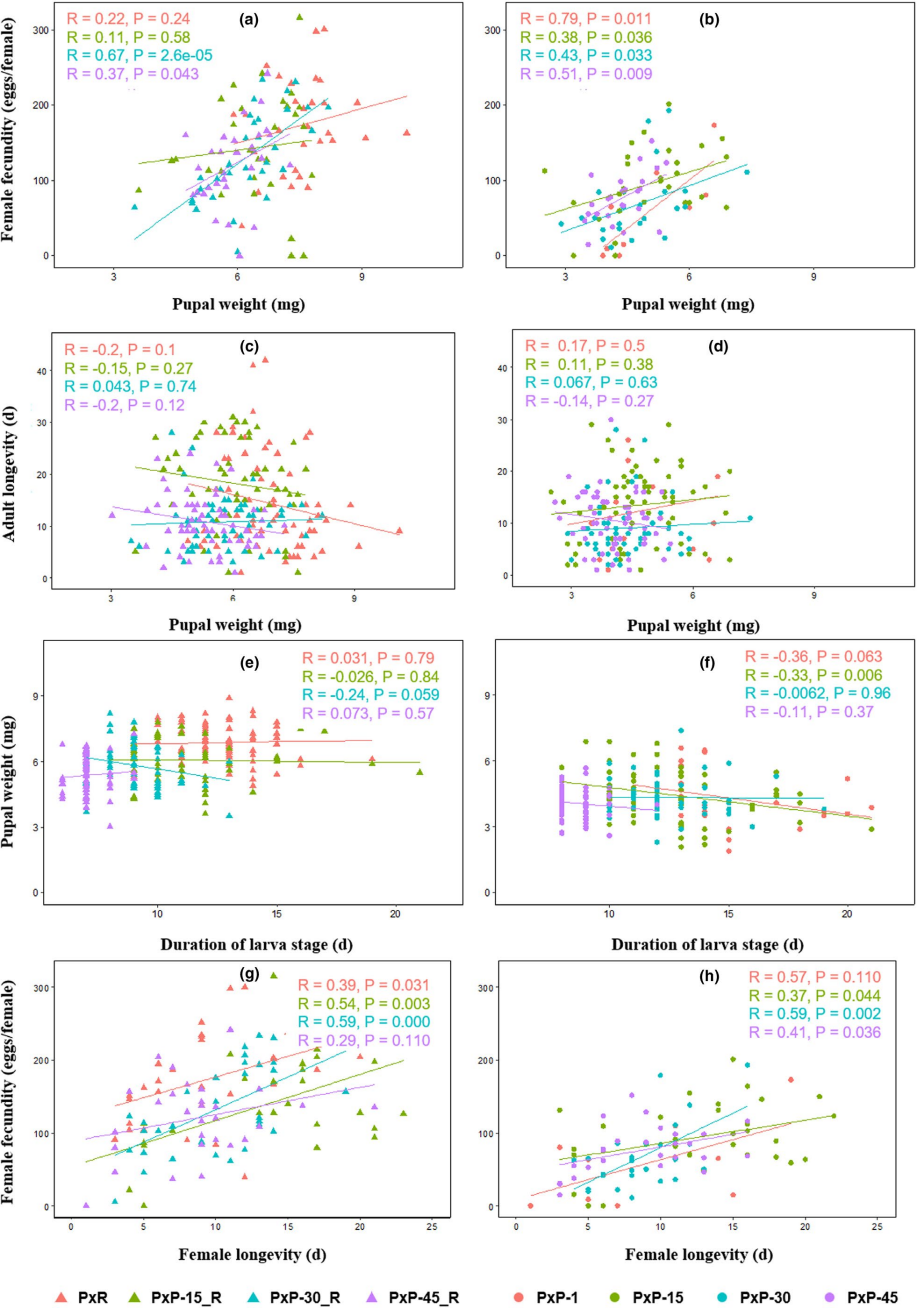
Correlations between pupal weight and female fecundity, pupal weight and duration of adult, duration of larval stage and pupal weight, and life span of female adults with female fecundity of PxP population (*Plutella xylostella* reared on pea) feeding on radish (a, c, e, g, respectively), and pea (b, d, f, h, respectively) in different generations. PxR, *P. xylostella* reared on radish; PxP‐1, PxP‐15, PxP‐30, and PxP‐45: the 1st, 15th, 30th, and 45th generations of PxP population; PxP‐15_R, PxP‐30_R, and PxP‐45_R: the 15th, 30th, and 45th generations of PxP population feeding on radish

Correlations between different fitness parameters are displayed in Figure 4. Significant positive correlations were found between pupal weight and female fecundity in pea‐fed populations (*p* <.05), but the correlations were not significant in PxR or in PxP‐15_R (Figure 4a,b). Pupal weight showed no correlations with adult longevity (Figure 4c,d). There were no correlations between the duration of the larval stage and pupal weight, with the exception of a negative correlation in the PxP‐15 generation (Figure 4e,f). In general, the female longevity was positively correlated with fecundity (*p* < .05) (Figure 4g,h).

## 4 DISCUSSION

Insect population parameters, such as survival, development time, and reproduction, are influenced by host plants and are key estimators to determine the adaptability of herbivorous insects to new host plants (Saeed et al., 2010). Higher adaptability is reflected by a higher progeny survival, shorter development time, and higher fecundity (Awmack & Leather, 2002; Roitberg et al., 2001). By providing *P. xylostella* with pea leaves as a sole food resource, we successfully established a PxP population and monitored the population dynamic parameters every 15 generations. Löhr and Gathu (2002) observed a larval survival rate of 2.4% when a *P. xylostella* crucifer strain (DBM‐C) was fed sugar snap peas. In our pilot study for the establishment of PxP population, the survival of the 1st generation fluctuated with pea variety (Purple Flower‐Soft Pod, Huazhen Purple Flower, Zhenbao, Zhaochun Sweet, Zhaochun Changshou, 863 Texuan, 578 Pink Flower, Texuan 13, Taizhong 11 and Taizhong 13) and leaf quality. Eventually, we found fresh leaves of “Purple Flower‐Soft Pod” cultivar were generally suitable with 17% *P. xylostella* survival rate of immature stages in the 1st generation. One possible reason is the bottom‐up effects of *Brassica* cultivars or genotypes on the performance of *P. xylostella* (Fathipour et al., 2019; Fathipour & Mirhosseini, 2017; Kianpour et al., 2014; Nikooei et al., 2015; Soufbaf et al., 2010). Löhr and Gathu (2002) found that the survival rate increased to 49.7% in the 4th generation. In the present study, the survival rate reached 60% by the 15th generation and thereafter remained stable, indicating quick adaptability of *P. xylostella* to peas. These differences may be caused by the genetic differentiation among populations in different parts of the world (Pichon et al., 2006).

Henniges‐Janssen et al. (2011) found a negative trend in mean generation time of the *P. xylostella* pea‐adapted strain (DBM‐P) on “Oregon Sugar Pod” cultivar when consistently observed for more than 50 generations. In the present study, we also found that as acclimation time increased, the mean generation time shortened further indicating high adaptability of *P. xylostella* to peas. Moreover, we found that the duration of the 4th instar was prolonged and that the mortality was high in the 1st generation of *P. xylostella* feeding on pea, compared with that of PxR. A future study investigating the transcriptome as it relates to adaptability will further explain these observations.

Reproduction is a critical biological indicator, especially in insects that typically produce hundreds of offspring that survive without parental protection (Harano, 2011). Host plant species and plant variety or genotype have been shown to influence fecundity in *P. xylostella*. Saeed et al. (2010) observed fecundity differences between *P. xylostella* fed different cruciferous vegetables, including cabbage, cauliflower, radish, turnip, mustard, and canola. Fecundity differences were found in different varieties, 440 eggs on Globe Master versus 102 eggs on Scarlet Ohara (Golizadeh et al., 2009b), 27 eggs on NSA2 versus 5 eggs on Red‐Rocky (Fathipour et al., 2019), and 61 eggs on Opera versus 8 eggs on PF (Nikooei et al., 2015). In the present study, the average fecundities of PxP feeding on pea were significantly lower than that of individuals feeding on radish in all generations. One possible explanation is that oviposition stimulators are low in pea. For example, glucosinolates can attract and stimulate *P. xylostella* to lay more eggs and shorten the prelaying period, but the pea plant has low glucosinolates content (Badenes‐Perez et al., 2014, 2020). Another possible explanation is that pea lacks sufficient nutrients for the reproduction of *P. xylostella*.

Darwin's fecundity advantage hypothesis suggests that larger females can reproduce more offspring (Afaq, 2013; Andersson, 1994; Darwin, 1874; Honěk, 1993). In the present study, PxP feeding on pea showed a significant positive correlation between pupal weight and fecundity, in accordance with insect species such as *Streblote panda* (Calvo & Molina, 2005), *Peregrinus maidis* (Wang et al., 2006), *Sitobion avenae*, *Rhopalosiphum padi*, and *Schizaphis graminum* (Hu et al., 2015). In contrast, a longer development period has been correlated with larger individuals (Nijhout et al., 2010; Teder et al., 2014). In addition, *Chilo suppressalis* (Huang et al., 2018) and *Ostrinia furnacalis* (Xia et al., 2019) showed a negative correlation between larval development time and pupal weight. However, no correlation was found between larval development time and pupal weight in our study. This variation in observations supports the conclusion of a previous study that development time and body mass could exhibit positive, negative, or zero correlation (Nijhout et al., 2010). Female fecundity and adult longevity are generally considered to have a “trade‐off” relationship: the cost of reproduction shortens adult longevity (Bell, 1986; Williams, 1966). This has been demonstrated in *Chorthippus brunneus* (De Souza Santos and & Begon, 1987), *Drosophila melanogaster* (Sambucetti et al., 2015), and *Helicoverpa armigera* (Thyloor et al., 2016). However, we observed a significant positive correlation between female longevity and fecundity (Figure 4).

In conclusion, we established a stable *P. xylostella* population on a marginal host plant upon long‐term acclimation. However, the observed fitness in PxP population feeding on pea was lower than that feeding on its original host, radish. Correlations between different fitness parameters were influenced by host plants. Our results may facilitate the prediction of pest behavior when the preferred host is absent.

## CONFLICT OF INTEREST

None declared.

## AUTHOR CONTRIBUTIONS

**Fei‐Ying Yang:** Conceptualization (equal); investigation (lead); methodology (lead); software (lead); visualization (lead); writing–original draft (lead); writing–review and editing (lead). **Jun‐Hui Chen:** Visualization (supporting); writing–original draft (supporting); writing–review and editing (supporting). **Qian‐Qian Ruan:** Investigation (supporting); methodology (supporting). **Bei‐Bei Wang:** Investigation (supporting); methodology (supporting). **Lu Jiao:** Investigation (supporting); methodology (supporting). **Qing‐Xuan Qiao:** Investigation (supporting); methodology (supporting). **Wei‐Yi He:** Conceptualization (equal); data curation (equal); funding acquisition (equal); project administration (lead); supervision (equal); writing–review and editing (equal). **Min‐Sheng You:** Conceptualization (lead); Data curation (lead); formal analysis (lead); funding acquisition (lead); project administration (equal); supervision (lead); validation (lead); writing–original draft (supporting); writing–review and editing (supporting).

## Data Availability

Empirical data have been archived in DataDryad: https://doi.org/10.5061/dryad.931zcrjkb.
